# Personality traits in established schizophrenia: aspects of usability and differences between patients and controls using the Swedish universities Scales of Personality

**DOI:** 10.3109/08039488.2016.1159331

**Published:** 2016-04-22

**Authors:** Tomas Fagerberg, Erik Söderman, J. Petter Gustavsson, Ingrid Agartz, Erik G. Jönsson

**Affiliations:** ^a^ Human Brain Informatics (HUBIN), Department of Clinical Neuroscience, Psychiatry Section, Karolinska Institutet and Hospital, Stockholm, Sweden; ^b^ Division of Psychology, Department of Clinical Neuroscience, Karolinska Institutet, Stockholm, Sweden; ^c^ NORMENT, KG Jebsen Centre for Psychosis Research, Institute of Clinical Medicine. Psychiatry section, University of Oslo, Norway; ^d^ Department of Psychiatric Research, Diakonhjemmet Hospital, Oslo, Norway

**Keywords:** Schizophrenia, personality, Swedish universities Scale of Personality (SSP)

## Abstract

**Background:** Personality is considered as an important aspect that can affect symptoms and social function in persons with schizophrenia. The personality questionnaire Swedish universities Scales of Personality (SSP) has not previously been used in psychotic disorder.

**Aims:** To investigate if SSP has a similar internal consistency and factor structure in a psychosis population as among healthy controls and if patients with psychotic disorders differ from non-psychotic individuals in their responses to the SSP.

**Methods:** Patients with psychotic disorders (*n* = 107) and healthy controls (*n* = 119) completed SSP. SSP scores were analyzed for internal consistency and case-control differences by Cronbach’s alfa and multiple analysis of covariance, respectively.

**Results:** Internal consistencies among patients were overall similar to that of controls. The patients scored significantly higher in seven (Somatic trait anxiety, Psychic trait anxiety, Stress susceptibility, Lack of assertiveness, Detachment, Embitterment, Mistrust) and lower in three (Physical trait aggression, Verbal trait aggression, Adventure seeking) of the 13 scales of the inventory. In three scales (Impulsiveness, Social desirability and Trait irritability) there was no significant difference between the scoring of patients and healthy controls.

**Conclusion:** The reliability estimates suggest that SSP can be used by patients with psychotic disorders in stable remission. Patients score higher on neuroticism-related scales and lower on aggression-related scales than controls, which is in accordance with earlier studies where other personality inventories were used.

## Background

Personality is considered as an important aspect, which can affect symptoms and social functioning in schizophrenia (1). Several studies have investigated personality in schizophrenia (2–5). These studies mainly used the inventories Tridimensional Personality Questionnaire (TPQ) (6,7), its successor Temperament and Character Inventory (TCI) (8) and the five factor model (FFM)-derived NEO personality inventories (PIs) NEO-FFI, NEO-PI and NEO-PI-R (9,10). The most consistent finding of these studies is a higher degree of Harm avoidance in the TPQ and TCI and Neuroticism in NEO questionnaires among patients with schizophrenia. Harm avoidance and Neuroticism are measuring tendencies to pessimism, worry, avoidance, shyness, fatigability, vulnerability to emotional instability and vulnerability to self-consciousness. Swedish universities Scales of Personality (SSP) is a personality inventory developed in Sweden (11). SSP is a further development of the inventory Karolinska Scales of Personality (KSP), where some of the scales, in contrast to the general FFM model, were developed to study vulnerability for psychopathic and schizoid traits (12) without aiming at covering the “whole personality”. For further background of the development of KSP and SSP, please see (11,12). SSP is composed of 13 different scales, i.e. Somatic trait anxiety (STA), Psychic trait anxiety (PsTA), Stress susceptibility (SS), Lack of assertiveness (LA), Detachment (D), Embitterment (E), Mistrust (M), Physical trait aggression (PhTA), Verbal trait aggression (VTA), Adventure seeking (AS), Impulsiveness (I), Social desirability (SD), and Trait irritability (TI) (11). Subjects scoring high in these scales are characterized by autonomic disturbances, restlessness and tense (STA), worrying, anticipating, and lack of self-confidence (PsTA), being easily fatigued and feeling uneasy when urged to speed up (SS), lack of ability to speak up and to be self-assertive in social situations (LA), avoiding involvement in others, being withdrawn and schizoid (D), un-satisfaction, blaming and envying others (E), suspiciousness and distrusting people’s motives (M), getting into fights, starting fights and hitting back (PhTA), getting into arguments and berating people when annoyed (VTA), avoiding routine and a need for change and action (AS), acting on the spur of the moment, non-planning and impulsive (I), socially confirming, friendliness, and helpfulness (SD), and irritability and lacking patience (TI) (11). SSP has been factor-analyzed into three major dimensions, of which neuroticism is one. SSP has not previously been used to investigate patients with schizophrenia or related psychosis disorder.

Previous studies have usually not published data on internal consistency in the samples of patients with psychotic disorders. The investigation of psychometric properties is important, because it is not necessarily true that a sub-group of the population with partly deviant cognitive abilities and symptomatic experiences and which are likely not to respond at any major degree in population-based questionnaires, would display a similar understanding of instruments tested and developed in the general population. Furthermore, with few exceptions previous studies have not analyzed sub-traits of the major dimensions. In order to shed further light into the personality of schizophrenia and related psychotic disorders, SSP was analyzed in a sample of patients with psychotic disorders and non-psychotic controls. We analyzed the internal consistency and the factor structure of SSP in patients with psychotic disorders when compared to data from healthy individuals or the general population. We also made a case-control evaluation in order to see how the different scales differed between patients with psychotic disorders and non-psychotic controls.

## Aims

We aimed to investigate if SSP is possible to use in a psychosis population and if patients with psychotic disorders differ in their responses to the SSP form from people without psychosis disorder.

## Materials and Methods

### Subjects

Patients with long term psychotic disorder and unrelated healthy controls who have been part of a study where clinical, genetic, neuropsychological and brain morphological aspects have been investigated (13–15), were asked to participate. Subjects were unrelated individuals of Caucasian origin recruited from psychiatric clinics (outpatients units) in Stockholm, between 1999 and 2003. They were diagnosed according to DSM-III-R and DSM-IV as previously described (16,17). Function was assessed by the Global assessment of functioning (GAF) scale (18). As a proxy for verbal intelligent quotient (IQ) the vocabulary part of Wechsler adult intelligence scales (WAIS) was used (19). Psychotic symptoms were assessed using the Scales for assessment of negative symptoms (SANS) and the Scales for assessment of positive symptoms (SAPS) (20,21). For a comprehensive overview of the patients’ consumption of antipsychotic drugs chlorpromazine equivalents were used (22). All subjects were given complete description of the study and participated after given informed written consent. The study was conducted in accordance with the Declaration of Helsinki and approved by the Swedish Data Inspection Board (Datainspektionen) and the Research Ethics Committee at Karolinska Institutet (2009/1403-31/1).

### Questionnaire

In connection with a research interview with a physician, patients filled in the personality inventory SSP. SSP is a self-report questionnaire and consist of 91 items grouped into 13 different scales. The patients must decide by agreeing with one of four possible answers; not true at all, not particularly good, agree somewhat, exactly right. The 13 different scales are; Somatic trait anxiety (STA), Psychic trait anxiety (PsTA), Stress susceptibility (SS), Lack of assertiveness (LA), Detachment (D), Embitterment (E), Mistrust (M), Physical trait aggression (PhTA), Verbal trait aggression (VTA), Adventure seeking (AS), Impulsiveness (I), Social desirability (SD), and Trait irritability (TI). Unrelated control subjects were asked to complete an extended version of KSP (KSP-196), used during the construction of SSP and other personality constructs (11,23). KSP-196 includes all of the 91 items contained in the SSP.

### Statistical analysis

Based on the 91 items that are common to the SSP and KSP-196, the 13 different personality scales was calculated according to the SSP manual. The subjects were divided into two groups: patients and healthy controls. Patients consisted of subjects who have been treated for psychosis. The controls consisted of unrelated non-psychotic control subjects.

Internal consistency was evaluated using Cronbach’s alfa (24). Factor analysis for personality traits was performed with varimax rotation in order to identify factors with eigenvalues >1. Principal axis factoring was used as the extraction method and the limit for factor loading was set at >0.45. In the case-control analyzes, to circumvent the issue of multiple testing and control for interaction effect between diagnosis and gender, as a first step multiple analysis of covariance (MANCOVA) was performed with diagnosis (patients *vs* controls) and gender as between-subjects factors and age as a covariate. As a second step analyzes of co-variance (ANCOVA) was performed for each SSP scale with diagnosis and gender as between-subject factors and age as a covariate. The statistical analysis was made using SPSS version 17.0.1 for Windows, IBM software.

## Results

Characteristics of the subjects are presented in Table 1. Among the 107 patients there were 35 (33%) women and among the 119 healthy subjects there were 48 (40%) women (Table 1). The mean age (SD; range) of male patients, female patients, male control subjects and female control subjects were 42.4 (8.98; 24-66), 41.1 (8.81; 24-61), 43.0 (8.19; 19-55), and 43.2 (7.74; 20-56) years, respectively (Table 3). There were no significant age or gender differences between patients and controls (Table 1). Patients were less educated, had a lower verbal IQ and a lower level of functioning (Table 1). Patients were diagnosed with schizophrenia (*n* = 82), schizoaffective disorder (*n* = 15), and psychosis not otherwise specified (*n* = 10). Their mean age at onset of illness was 24.3 years.

**Table 1.** Characteristics of patients and controls.

	Patients (*n* = 107)	Controls (*n* = 119)	*P*-value
Gender (*n*, women/men)	35/72	48/71	NS^a^
Age (year)	41.9 (8.9)	43.1 (8.0)	NS^b^
Education (year)	12.7 (3.0)	14.2 (2.8)	*P* < 0.001^b^
WAIS verbal IQ	87.9 (20.8)	102.4 (15.9)	*P* < 0.001^b^
GAF	48.8 (9.4)	85.8 (7.3)	*P* < 0.001^b^
Diagnosis – schizophrenia (*n*)	82	NA	
Diagnosis – schizoaffective disorder (*n*)	15	NA	
Diagnosis – psychosis NOS (*n*)	10	NA	
Age at onset of illness (year)	24.3 (5.0)	NA	
SANS composite score	30.9 (21.6)	NA	
SAPS composite score	11.2 (9.0)	NA	
Medication (mg, CPZ-equivalents)	252.9 (233.8)	NA	
Medication – no antipsychotics (*n*)	7	NA	
Medication – 2nd gen antipsychotics (*n*)	47	NA	
Medication – 1st gen antipsychotics (*n*)	46	NA	
Medication – 1st and 2nd gen antipsychotics (*n*)	7	NA	

NS: not significant; NA: not applicable; WAIS: Wechsler adult Intelligence Scales; IQ: intelligent quotient; GAF: Global Assessment of Functioning; SANS: Scale for the Assessment of Negative Symptoms; SAPS: Scale for the Assessment of Positive Symptoms; CPZ: chlorpromazine; gen: generation; NOS: not otherwise specified. All values in mean (standard deviation) except for distribution of gender, diagnosis and medication.

Missing data (patients/controls): Education (2/2), WAIS vocabulary (30/38), GAF (1/0), SANS (29), SAPS (29).

^a^χ^2^-test. ^b^Unpaired two-sided *t*-test.

Internal consistency evaluation revealed Cronbach’s alfa coefficients between 0.67–0.81 and 0.69–0.86 among patients and controls, respectively, with three exceptions: among patients Somatic Trait Anxiety and Social Desirability yielded alfa-levels of 0.59 and 0.55, respectively, and among controls the alfa-level of Social Desirability was 0.52 (Table 2). Similarly to the Swedish normative study (11) the scale with lowest internal consistency was Social Desirability. In particular item 86 (“It has happened that I have to lie to get away from something I did not want to do”) showed a low internal consistency with the other items of Social Desirability. Still, the coefficients also among patients were generally close to those obtained in two normative studies (11,25) and usually in the range which is considered acceptable (Table 2).

**Table 2.** Swedish Scales of Personality (SSP) internal consistency data given as Cronbach’s alfa for psychotic patients (*n* = 107) and control subjects (*n* = 119). Data from the Swedish normative study (*n* = 741) and an Estonian study of healthy subjects (*n* = 529) is shown for comparison (11,25).

SSP scale	Patients	Controls	(11)	(25)
Neuroticism	0.82	0.89	–	–
Aggressiveness	0.62	0.71	–	–
Extraversion	0.54	0.46	–	–
Somatic trait anxiety	0.59	0.80	0.75	0.75
Psychic trait anxiety	0.79	0.86	0.82	0.82
Stress susceptibility	0.67	0.80	0.74	0.73
Lack of sssertiveness	0.75	0.77	0.78	0.64
Impulsiveness	0.70	0.69	0.73	0.62
Adventure seeking	0.81	0.84	0.84	0.80
Detachment	0.71	0.80	0.77	0.58
Social desirability	0.55	0.52	0.59	0.66
Embitterment	0.67	0.77	0.75	0.73
Trait irritability	0.73	0.78	0.78	0.85
Mistrust	0.78	0.84	0.78	0.76
Verbal trait aggression	0.78	0.71	0.74	0.78
Physical trait aggression	0.75	0.85	0.84	0.84

**Table 3.** Swedish universities Scales of Personality (SSP) raw scores (mean [standard deviation]) in patients with psychotic illness (Pat) and non-psychotic controls (Con). Data is shown by diagnosis (pat *vs* con) in all subjects, gender in all subjects, gender among patients and gender among controls. *P*-values stem from analyzes of covariance assessing diagnosis, gender and diagnosis * gender with age as a covariate.

		*N*	Age (year)	STA	PsTA	SS	LA	I	AS
ALL	Pat	107	41.93 (8.91)	2.06 (0.53)***	2.48 (0.65)***	2.51 (0.54)***	2.52 (0.61)***	2.26 (0.58)	2.27 (0.63)*
	Con	119	43.06 (7.98)	1.56 (0.52)	1.68 (0.54)	1.84 (0.47)	1.91 (0.48)	2.30 (0.45)	2.47 (0.53)
ALL	Men	143	42.65 (8.58)	1.74 (0.54)*	2.04 (0.74)	2.16 (0.62)	2.20 (0.65)	2.22 (0.51)*	2.38 (0.60)
	Women	83	42.30 (8.22)	1.89 (0.64)	2.10 (0.68)	2.16 (0.58)	2.19 (0.59)	2.39 (0.52)	2.37 (0.57)
PAT	Men	72	42.35 (8.99)	2.06 (0.47)	2.49 (0.64)	2.51 (0.56)	2.53 (0.61)	2.24 (0.58)	2.26 (0.64)
	Women	35	41.06 (8.81)	2.06 (0.65)	2.47 (0.68)	2.51 (0.50)	2.48 (0.63)	2.29 (0.59)	2.30 (0.62)
CON	Men	71	42.96 (8.19)	1.43 (0.41)	1.59 (0.53)	1.80 (0.45)	1.87 (0.50)	2.20 (0.41)	2.50 (0.53)
	Women	48	43.20 (7.74)	1.76 (0.61)	1.82 (0.52)	1.90 (0.48)	1.98 (0.45)	2.46 (0.45)	2.42 (0.54)
		*N*	D	SD	E	TI	M	VTA	PhTA
ALL	Pat	107	2.41 (0.58)***	2.75 (0.49)	2.23 (0.57)***	2.22 (0.57)	2.14 (0.59)***	1.95 (0.61)*	1.73 (0.53)**
	Con	119	1.92 (0.47)	2.82 (0.33)	1.60 (0.43)	2.21 (0.47)	1.68 (0.47)	2.12 (0.46)	1.91 (0.55)
ALL	Men	143	2.23 (0.58)*	2.78 (0.42)	1.90 (0.60)	2.21 (0.51)	1.94 (0.61)	2.04 (0.55)	1.89 (0.54)*
	Women	83	2.01 (0.55)	2.79 (0.40)	1.90 (0.58)	2.23 (0.54)	1.83 (0.52)	2.05 (0.52)	1.72 (0.53)
PAT	Men	72	2.45 (0.60)	2.76 (0.51)	2.22 (0.56)	2.24 (0.58)	2.19 (0.63)	1.97 (0.63)	1.79 (0.54)
	Women	35	2.32 (0.53)	2.72 (0.45)	2.23 (0.60)	2.19 (0.57)	2.05 (0.52)	1.93 (0.56)	1.60 (0.50)
CON	Men	71	2.01 (0.46)	2.80 (0.32)	1.57 (0.43)	2.18 (0.43)	1.68 (0.48)	2.11 (0.45)	1.99 (0.54)
	Women	48	1.79 (0.46)	2.83 (0.35)	1.66 (0.43)	2.26 (0.52)	1.68 (0.46)	2.14 (0.47)	1.80 (0.55)
		*N*	Neuroticism	Aggressiveness	Extraversion				
ALL	Pat	107	2.32 (0.43)***	2.16 (0.38)**	2.31 (0.36)				
	Con	119	1.71 (0.39)	2.27 (0.28)	2.23 (0.30)				
ALL	Men	143	2.00 (0.53)	2.23 (0.34)	2.28 (0.33)				
	Women	83	2.01 (0.47)	2.19 (0.33)	2.25 (0.34)				
PAT	Men	72	2.33 (0.43)	2.19 (0.40)	2.32 (0.36)				
	Women	35	2.30 (0.43))	2.11 (0.36)	2.29 (0.38)				
CON	Men	71	1.65 (0.39)	2.27 (0.27)	2.23 (0.30)				
	Women	48	1.80 (0.38)	2.26 (0.31)	2.20 (0.30)				

*p < 0.05.

**p < 0.01.

***p < 0.001; *N*: number; STA: Somatic trait anxiety; PsTA: Psychic trait anxiety; SS: Stress susceptibility; LA: Lack of assertiveness; I: Impulsiveness; AS: Adventure seeking; D: Detachment; SD: Social desirability; E: Embitterment; TI: Trait irritability; M: Misstrust; VTA: Verbal trait aggression; PhTA: Physical trait aggression.

To get an insight into how patients with psychotic disorders answer to the questionnaire, although the number of individuals was too small for robust evaluations, we also performed pilot factor analyzes. These showed as anticipated a three factor model (Table 4). Among patients factor 1 (Neuroticism) was similar to the Swedish normative study (11), whereas factor 2 (Aggressiveness) also included high loadings from the scales Impulsiveness, Adventure Seeking and Mistrust, which in the normative study mainly loaded on factor 3 (Extraversion), factor 3 and factor 1, respectively (11). In patients the third factor consisted of the scales Detachment, i.e. similar to the normative study (11), and Social Desirability, which loaded on the Aggressiveness factor in the normative study (11). Among controls the loading was as in the normative study (11) with the exception of the scale Detachment, which mainly loaded on the Neuroticism factor, similar to another recent Swedish study (26).

**Table 4.** Swedish universities Scales of Personality (SSP) factor structure in patients with psychotic illness, non-psychotic controls and in the Swedish normative sample (11).

	Patients			Controls			Normative sample		
	Loading on			Loading on			Loading on		
	Factor 1	Factor 2	Factor 3	Factor 1	Factor 2	Factor 3	Factor 1	Factor 2	Factor 3
STA	**0.66**	0.28	0.08	**0.70**	0.17	0.28	**0.73**	0.19	0.25
PsTA	**0.85**	0.07	−0.05	**0.91**	−0.02	0.06	**0.89**	0.02	−0.08
SS	**0.69**	0.10	−0.33	**0.86**	0.02	−0.04	**0.80**	0.16	−0.16
LA	**0.83**	−0.13	0.00	**0.77**	−0.35	−0.11	**0.72**	−0.27	−0.24
I	0.30	**0.55**	0.30	0.40	0.13	**0.74**	0.14	0.23	**0.73**
AS	−0.00	**0.59**	**0.47**	−0.11	0.28	**0.76**	−0.09	0.10	**0.82**
D	0.40	−0.08	**−0.74**	**0.60**	0.22	**−0.46**	0.35	0.40	**−0.52**
SD	0.15	−0.06	**0.77**	−0.07	**−0.65**	0.31	−0.10	**−0.69**	0.21
E	**0.67**	0.34	0.12	**0.81**	0.16	0.21	**0.64**	0.41	0.29
TI	0.31	**0.74**	−0.20	0.38	**0.69**	0.29	0.43	**0.64**	0.16
M	**0.52**	**0.55**	−0.10	**0.75**	0.31	−0.03	**0.53**	0.44	−0.04
VTA	−0.06	**0.84**	0.03	0.04	**0.79**	0.26	0.03	**0.80**	0.25
PhTA	0.07	**0.81**	−0.01	−0.03	**0.67**	0.19	−0.03	**0.70**	0.22

STA: Somatic trait anxiety; PsTA: Psychic trait anxiety; SS: Stress susceptibility; LA: Lack of assertiveness; I: Impulsiveness; AS: Adventure seeking; D: Detachment; SD: Social desirability; E: Embitterment; TI: Trait irritability; M: Misstrust; VTA: Verbal trait aggression; PhTA: Physical trait aggression; Bold values indicates loading factor > 0.45.

MANCOVA estimates showed effects of age (Wilk’s lambda = 0.868, *p* = 0.004), diagnosis (Wilk’s lambda = 0.574, *p* < 0.001) and gender (Wilk’s lambda = 0.849, *p* = 0.001). However, there was no interaction effect of diagnosis and gender (Wilk’s lambda = 0.950, *p* = 0.614). In post-hoc analyzes ANCOVAs was performed for each of the SSP scales. For ten of the scales, i.e. Somatic trait anxiety (F = 42.4, *p* < 0.001, patients’ scores [pat] higher than [>] controls’ scores [con]), Physical trait anxiety (F = 86.5, *p* < 0.001, pat > con), Stress susceptibility (F = 86.2, *p* < 0.001, pat > con), Lack of assertiveness (F = 54.9, *p* < 0.001, pat > con), Adventure seeking (F = 6.7, *p* = 0.010, con > pat), Detachment (F = 46.6, *p* < 0.001, pat > con), Embitterment (F = 70.6, *p* < 0.001, pat > con), Mistrust (F = 36.2, *p* < 0.001, pat > con), Verbal trait aggression (F = 6.0, *p* = 0.015, con > pat), and Physical trait aggression (F = 7.5, *p* = 0.007, con > pat), patients and controls scored significantly different, whereas for three (Impulsiveness [F = 0.8, *p* = 0.371], Social desirability [F = 1.7, *p* = 0.194], Trait irritability [F = 0.0, *p* = 0.985]) no significant differences were found. Gender effects were found for four of the scales (Somatic trait anxiety [F = 6.3, *p* = 0.013], Impulsiveness [F = 5.6, *p* = 0.019], Detachment [F = 5.2, *p* = 0.023] and Physical trait aggression [F = 6.6, *p* = 0.011]) (Table 3). Inspection of Table 3 revealed that this latter effect was mainly explained by gender differences in the control group only for Somatic trait anxiety (women > men), Impulsiveness (women > men) and Detachment (men > women), whereas in Physical trait aggression (men > women) there was a tendency for a gender difference also among patients.

We also performed analyzes with regard to higher-order factors as they appeared in the Swedish normative sample, i.e. Neuroticism, Aggressiveness, and Extraversion (11). MANCOVA estimates showed effects of age (Wilk’s lambda = 0.935, *p* = 0.002) and diagnosis (Wilk’s lambda = 0.610, *p* < 0.001) but not gender (Wilk’s lambda = 0.986, *p* = 0.382) or diagnosis x gender (Wilk’s lambda = 0.984, *p* = 0.319). In post-hoc analyzes ANCOVAs was performed for each of the SSP factors. In the Neuroticism factor patients scored significantly higher than controls (F = 106.31, *p* < 0.001), whereas in the Aggressiveness factor patients scored lower than controls (F = 7.77, *p* = 0.006). No significant findings emerged in the comparison of patients and controls in the Extraversion factor (F = 2.08, *p* = 0.151).

As measurements for psychotic symptoms (SANS, SAPS) were lacking among all controls and none of the controls used antipsychotic medication, we used correlations among patients with these measurements to get an idea of how these variables were associated with the SSP-subscales and factors. After Bonferroni-correction for multiple testing, there was association between SANS composite scores and SSP sub-scale I (*r* = −0.415, *p* = 0.0026) and SSP factors Aggressiveness (*r*=−0.347, *p* = 0.0054) and Extraversion (*r* = −0.315, *p* = 0.015). There was no association between SAPS scores and SSP sub-scales or SSP factors. Among subjects with antipsychotic medication there was a correlation between antipsychotic equivalents and SSP VTA (*r* = −0.323, *p* = 0.009) and SSP factor Aggressiveness (*r* = −0.268, *p* = 0.016). These results suggest that symptom load or antipsychotic medication do not majorly influence the results.

## Discussion

The main findings of the present study were two: first, with respect to internal consistency and factor structure, SSP seemed to be a psychometrically sound instrument investigating personality among patients with psychotic disorders in a stable phase of their illness. Secondly, patients with psychotic disorders scored significantly different in comparison with non-psychotic subjects on the majority of the investigated SSP-scales.

### Is response to personality inventories reliable among patients with psychotic disorders?

Could patients with psychotic disorders give reliable answers to the claims assessed in personality inventories? Internal consistency data showed similar patterns in patients, although mostly with somewhat lower values, compared to healthy individuals (Table 2). Unfortunately, the number of subjects in the factor structure evaluation was too low to make firm conclusions. However, the pilot investigation showed an overall factor structure reasonably similar in patients as in the Swedish normative sample, suggesting that patients with psychotic disorders have a general understanding of the questions in the SSP form similar to non-psychotic individuals. We are only aware of two studies, which have reported internal consistency of personality among patients with psychotic disorders filling in TPQ, TCI or NEO questionnaires. In one study 122 patients with paranoid schizophrenia were analyzed with the TCI, with Cronbach’s alfa values ranging from 0.16 to 0.77 for the four temperaments (Harm Avoidance 0.77, Novelty Seeking 0.66, Reward Dependance 0.30, Persistence 0.16) and 0.84 to 0.89 for the three characters (27). Other researchers analyzed 91 patients with schizophrenia, schizophreniform disorder, schizoaffective disorder, delusional disorder and psychosis not otherwise specified at two occasions three years apart with NEO-FFI and reported internal consistency between 0.61 and 0.91 for the five personality factors, and lower Chronbach’s alfa values in eight of ten analyzes among patients than in a healthy control group (28). This suggests that the use of SSP as a questionnaire rating individual differences in personality among patients with psychotic disorders has about similar psychometric properties as TCI and NEO.

The response patterns between patients and controls did not always differ from that of the healthy subjects. Of particular interest is the scale Social desirability, which is developed from a KSP-scale, which initially was included as a lie-scale for detecting abusive answers. In this scale there was no significant difference between the answers from the patients and healthy controls, similar to previous studies using the corresponding KSP scale (29,30). This further suggests that patients in a stable phase of their illness can give reliable answers.

In the current study, the patients were in stable conditions. No one was so ill that she or he needed to be treated in hospital. To assess whether patients during a relapse of their disorder can give reliable answers, investigations under such conditions has to be performed.

### KSP and schizophrenia

We are only aware of two studies using KSP in schizophrenia (29,30). The main finding in the most recent study, including 23 patients with schizophrenia and 14 control subjects, was differences between patients and controls in ten out of 15 scales. Patients scored substantially higher (surviving Bonferroni-corrected multiple testing) for the neuroticism-related scales Somatic anxiety (corresponding to SSP-scale STA), Psychic anxiety (PTA), Muscular tension, Psychastenia (SS), and Suscpicion (M), and lower for Socialization (inverse E), but also higher in Detachment (D), i.e. similar to the present study (Table 3). In addition, for the scales Irritability (TI), Guilt and Inhibition of aggression (LA) less convincing associations were reported. In the present study, patients scored higher in the scale LA, but not TI (Table 3). For five scales, i.e. Impulsiveness (I), Monotony avoidance (AS), Verbal aggression (VTA), Indirect aggression and Social desirability (SD) no significant case-control differences was found, whereas in the present study patients scored significantly lower in AS and VTA than controls (Table 3). The same pattern was evident also in the previous study, but did not reach statistical significance, probably as a result of lower power. Overall, the results of the present and the previous study are concordant in most respects.

Rasmussen and Levander (30) investigated 13 aggressive patients with schizophrenia treated in a forensic hospital, 13 non-aggressive patients with schizophrenia treated in non-forensic psychiatric wards and 13 controls, consisting of staff in the forensic hospital. The non-aggressive patients, reflecting the vast majority of patients with schizophrenia, scored higher in KSP Somatic anxiety, Psychic anxiety, Muscular tension, Psychastenia, Inhibition of aggression and Detachment and lower in Socialization than controls. There was a trend for association in Suspicion with higher scores in both non-aggressive and aggressive patients. Aggressive patients scored higher in Psychastenia and lower in Socialization, but did otherwise score similar to the controls. For the scales Impulsiveness, Monotony avoidance, Indirect aggression, Verbal aggression, Irritability, Guilt and Social desirability, no significant differences was found between either of the patient groups and controls. The results of the present study are thus in considerable agreement also with the study of Rasmussen and Levander with regard to the non-aggressive patients, but partly deviant with the aggressive patient group, who in contrast to the non-aggressive patients tended to score “normal” in the neuroticism-related subscales Somatic anxiety, Psychic anxiety, Muscular tension, and Inhibition of aggression (30).

### TPQ, TCI and schizophrenia

Previous studies, which have been using the personality inventories TPQ and TCI, have shown that patients with schizophrenia score higher in the scale Harm avoidance (4,31–35). Harm avoidance corresponds mainly to the SSP-scales Somatic trait anxiety, Psychic trait anxiety, Stress susceptibility and Lack of assertiveness (36). In the present study the patients score higher in these scales. The present results are thus in accordance with earlier results with the TCI inventory with regard to Harm avoidance (3,4,35,37–42).

Using TCI it has been noted that patients with schizophrenia in some (4,37,39) but not all (35,38,40–42) studies score lower in the scale Novelty seeking. Novelty seeking corresponds to the SSP scales Impulsiveness and Adventure seeking (36). In the present study the patients scored lower than healthy subjects in Adventure seeking, but there was no significant difference between patients and healthy subjects in the scale Impulsiveness. The results support earlier studies about Novelty seeking in psychotic disorder.

Concerning the TCI-scales Reward dependance and Persistence previous studies have shown that persons with schizophrenia score lower than healthy controls (4). Reward dependance corresponds to the inverse SSP-scale Detachment. In the present study, patients scored higher in that scale than healthy subjects. The results in these respects are thus in line with previous research.

### NEO and schizophrenia

Other studies have used the personality inventories NEO-FFI, NEO-PI and NEO-PI-R. In these studies patients with schizophrenia-like psychotic disorder scored higher in the personality scale Neuroticism (1,2,5,43–46). Neuroticism in the NEO inventory is reflected by the SSP-scales Somatic trait anxiety, Lack of assertiveness and inverse Detachment (25). In the current study patients scored higher on these scales than healthy subjects. The present results are thus substantially consistent with previous findings with the NEO inventories.

In previous studies with the NEO-inventories, patients with schizophrenia scored lower than the healthy subjects in the scale Extraversion (5), which in SSP closest match to the scales Adventure seeking, inverse Lack of assertiveness and inverse Detachment (5). In the present study patients scored lower in Adventure seeking and higher in Lack of assertiveness and Detachment, which is in line with previous findings with the NEO-inventories (2,5).

In previous studies with the NEO-inventories, the results concerning the scale Agreeableness indicated that persons with schizophrenia scored lower than healthy subjects (5,46). Aspects of Agreeableness correspond in SSP to the scales Social desirability, inverse Verbal trait aggression and inverse Physical trait aggression (5). In the present study patients scored slightly lower than healthy subjects in Verbal trait aggression and Physical trait aggression. There was however no significant difference between patients and healthy subjects in the SSP-scale Social desirability. The present study cannot confirm the earlier findings in these parts. Varying ethnicity in the different studies may be a possible explanation for the discrepant results.

### Personality in other psychiatric conditions

In previous studies with the TCI inventory, it has been demonstrated that persons with depressive symptoms (47), patients diagnosed with depression (48–50), patients with bipolar disorder (50–52) and patients with panic disorder (53) scored higher than healthy subjects in Harm Avoidance, a scale which essentially corresponds to Neuroticism in the NEO inventories.

In a meta-analysis investigating studies where the NEO inventories has been used to examine differences between patients with a range of non-psychotic disorders, such as depression, dysthymia, generalized anxiety disorder, post traumatic stress disorder, panic disorder, agoraphobia, social phobia, specific phobias, obsessive compulsive disorder, and alcohol- and drug dependance showed that all patient groups scored higher in the scale Neuroticism and tended to estimate lower in the scale Extraversion (54).

In one study where SSP was used to estimate personality in couples who underwent in vitro fertilization, individuals with a psychiatric diagnosis (preferably depression and anxiety disorders) scored higher on neuroticism-related scales than individuals who where healthy (55). Another study used SSP to assess the relationship between personality factors and postpartum depression, and found associations between neuroticism-related scales and depressive symptoms at six weeks and six months postpartum (26). One study used SSP to investigate panic disorder and found that patients score higher in neuroticism-related scales than healthy subjects (56). In a study where patients with severe health anxiety were treated with internet-based psychotherapy their elevated scores in several neuroticism-related SSP scales were reduced after treatment (57). This indicates that high scores in scales related to neuroticism is a general finding among patients with psychiatric disorders and possibly a general marker of psychopathology.

### Why do patients and healthy subjects estimate different?

Patients with psychotic disorders and control subjects differ in several aspects: The patients almost always use antipsychotic drugs in contrast to healthy subjects. It is reasonable to believe that extrapyramidal side effects could contribute to the response results in some items, for example parts of those covering anxiety and detachment. When we analyzed relationships between antipsychotic equivalents and SSP sub-scales among patients there were no stable correlations with one exception: in VTA higher antipsychotic doses were associated with lower scores of verbal aggression. This suggests that antipsychotic medication do not in any major way influence the scoring of the personality traits in SSP. This is in agreement with the results from previous studies of other patient groups, where antipsychotic drugs are not the main treatment, and where the patients still estimates higher on neuroticism-related scales, further arguing against antipsychotic drug treatment as a major cause of the case-control differences found in the current study, and rather suggesting that neuroticism is a common marker for a wide range of psychopathology. In order to be able to exclude the effects of antipsychotic drug treatment it would be necessary to conduct a study in which patients with recent onset psychosis fill in personality questionnaires before drug treatment.

### Limitations

Among the limitations of the current study is that both patients and healthy subjects consist of persons who have agreed to participate in a partially strenuous biological research. The group is thus not representative of either patients with psychotic disorders or healthy subjects. On the other hand, the presence of a non-psychotic control group who were also involved in the same biological research as the patients may be seen as strength of the study. Another limitation is the relatively small sample size, excluding robust investigations of the factor structure. The majority of patients were treated, in contrast to the control subjects, with antipsychotic drugs, which may affect the results.

SSP, which is used in the current study, is not identical with the personality inventories used in earlier studies. This makes it more difficult to compare the present study with previous studies. SSP, however, captures most of the personality traits that are reflected in other instruments and may have particular advantages. For example, some scales in SSP and its predecessor appear to have biological relevance (58–60).

## Conclusions

Reliability estimates suggest that patients with psychotic disorders in stable remission can adequately fill in the personality inventory SSP. Patients scored higher on neuroticism-related scales and lower on aggression-related scales than controls, which is in accordance with earlier studies where other personality inventories were used.
